# Resolving Issues of Content Uniformity and Low Permeability Using Eutectic Blend of Camphor and Menthol

**DOI:** 10.4103/0250-474X.59543

**Published:** 2009

**Authors:** M. C. Gohel, S. A. Nagori

**Affiliations:** Department of Pharmaceutics and Pharmaceutical Technology, L. M. College of Pharmacy, Navrangpura, Ahmedabad-380 009, India

**Keywords:** Captopril, co-grinding, content uniformity, eutectic mixture, permeability, short term stability study

## Abstract

The aim of present study were to arrest the problem of content uniformity without the use of harmful organic solvent and to improve *ex vivo* permeability of captopril, a low dose class III drug as per biological classification system. Eutectic mixture of camphor and menthol was innovatively used in the work. Captopril solution in eutectic mixture was blended with Avicel PH 102 and then the mixture was blended with mannitol in different ratios. Formulated batches were characterized for angle of repose and Carr's index. A selected batch was filled in hard gelatin capsule. Tablet dosage form was also developed. Capsules and tablets were characterized for *in vitro* drug release in 0.1N HCl. Additionally, the captopril tablets were analyzed for content uniformity and *ex vivo* drug permeation study using rat ileum in modified apparatus. The measurement of angle of repose and Carr's index revealed that the powder blend exhibited good flow property and compressibility. The captopril capsules and tablets exhibited immediate drug release in 0.1 N HCl. The captopril tablets passed content uniformity test as per IP 1996. *Ex vivo* permeation of captopril, formulated with eutectic mixture, was faster than control. The permeation was increased by 15% at the end of 3 h. Tablets and capsule exhibited reasonable short term stability with no considerable change in performance characteristics.

Formulation development is required to be carried out by keeping the FDA's and patients requirements in mind. The most debated requirements are content uniformity and permeability. A Biopharmaceutical Classification System (BCS) class III low dose drug requires attention from both the perspectives. The simplest mean of addressing both problems concurrently is to blend drug solution with excipients. The use of classical organic solvents is not favoured due to environmental considerations. An effort is made in the present work to use edible excipients to address the critical issues.

A eutectic mixture is a mixture of two or more solid compounds at a composition that has the lowest melting point. Camphor and menthol forms a hydrophobic eutectic mixture. Camphor is a terpenoid with the chemical formula C_10_H_16_O. Camphor has a high melting point (180°) and is a highly volatile substance with strong pine-like odor that sublimes even at room temperature and pressure. Camphor water was an official Pharmacopoeial product[1]. Menthol is chemically (1R,2S,5R)-5-methyl-2-(1-methyethyl)-cyclohexanol). Its molecular weight is 156 and melting point is 42°. Menthol is widely used in pharmaceuticals, confectionery and toiletry products as a flavoring agent or odor enhancer.

In the present study, eutectic mixture of camphor and menthol was used as a solvent for captopril. The solution was adsorbed on Avicel PH 102 to overcome the problem of content uniformity and improve permeability of BCS class III low dose drug without infringing existing patents. BCS class III drugs have good solubility but poor permeability[2]. Camphor and menthol are powerful penetration enhancers[3]. Hence, presence of traces of eutectic mixture of camphor and menthol can significantly improve the bioavailability of Biological Classification System (BCS) class III drugs.

Captopril (1-[(2S)-3-mercapto-2-methylprop-ionyl]-L-proline), was chosen as a model BCS class III drug. Captopril became generic in the US in 1996 as a result of the end of market exclusivity for Bristol-Myers Squibb. It is white to off-white crystalline powder with slight sulphurous odour. Aqueous solubility, log P and clog P values of captopril are 160 mg/ml, 0.23 and 0.88, respectively. Captopril demonstrates excellent clinical effectiveness in the treatment of hypertension by inhibiting angiotensin converting enzyme (ACE). In present study, immediate release formulations of captopril were developed.

## MATERIALS AND METHODS

Captopril, microcrystalline cellulose (Avicel PH 102) and sodium starch glycolate were received as gift samples from Zydus Cadila (Ahmedabad, India). Camphor and menthol were purchased from Gem Corporation (Ahmedabad, India) and Shreeji Pharma International (Ahmedabad, India), respectively. Mannitol was purchased from S. D. Fine Chemicals (Boisar, India). Polyvinylpyrrolidone (PVP K30) and potassium dichromate were purchased from Laser Laboratories (Ahmedabad, India). Cab-O-Sil M5 was received as a gift from Cabot Sanmar Pvt. Ltd. (Ahmedabad, India). The other chemicals and reagents were of analytical grade.

### Determination of eutectic composition of camphor and menthol:

Camphor and menthol were mixed in different ratios at 35±2° for 15 min in a mortar and pestle (Table 1). The undissolved solids, if any, were carefully scraped with the help of a spatula and weighed. The presence of only one phase, i.e. liquid, indicate eutectic composition. The composition and results are shown in Table 1.

**TABLE 1:** DETERMINATION OF EUTECTIC COMPOSITION

Ratio of menthol: camphor	Results (n=3)

	Percentage undissolved solids
10:90	95±2.5
20:80	64.4±2.5
30:70	50±2.5
40:60	16±2.5
50:50	0
60:40	0
70:30	0
80:20	0
90:10	0

### Determination of solubility of captopril in eutectic mixture of camphor and menthol:

An excess amount of captopril was added to 35 ml eutectic mixture consisting of equal amount of camphor and menthol and stirred at 100 rpm on a magnetic stirrer (Remi Electronics, Ahmedabad, India) for 30 min at 35±2° in a closed vessel. The mixtures were filtered through a 0.2 μm millipore filter. The weight of undissolved solid was recorded.

### Formulation:

Captopril and PVP K30 were dissolved in 12 ml of eutectic mixture consisting of equal amount of menthol and camphor in a closed container. The resulting solution of captopril was adsorbed on Avicel PH 102. The wet mass was passed through 20# mesh screen (850 μm opening) and dried at 92±2° in a tray dryer. The dried granules (20#, batch B1, Table 2) were mixed with sodium starch glycolate and characterized for angle of repose and Carr's index.

**TABLE 2:** COMPOSITION OF VARIOUS FORMULATED BATCHES B1-B9

Batch code	Composition						
	
	Captopril (g)	PVP K30 (g)	Eutectic mixture (ml)	Avicel PH 102 (g)	Sodium starch glycolate (g)	Cab-O-Sil (g)	Mannitol (g)
B1	2.4	0.6	12	11	1	0	0
B2	2.4	0.6	12	11	1	0.075	0
B3	2.4	0.6	12	11	1	0.075	0
B4	2.4	0.6	12	11	1	0.1125	0
B5	2.4	0.6	12	11	1	0.15	0
B6	2.4	0.6	12	11	1	0	3.75
B7	2.4	0.6	12	11	1	0	7.5
B8	2.4	0.6	12	11	1	0	11.25
B9	2.4	0.6	12	11	1	0	15

Batch B10 was prepared using powder blend of batch B9 (equivalent to 12.5 mg captopril). Batch B11 was prepared by compressing powder blend of batch B9 (equivalent to 12.5 mg captopril). Batch B12 was prepared using 95% alcohol in place of eutectic mixture of camphor and menthol. Batch B13 tablets were prepared using physical mixture of captopril, Avicel PH 102, mannitol and sodium starch glycolate in concentration equivalent to batch B9.

**Fig. 1:**
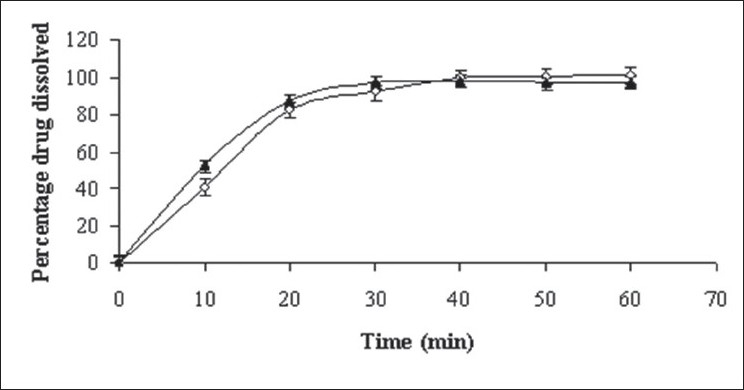
*In vitro* drug release of formulated batches B10 and B11 Batch B10 (–▲–) and batch B11 (
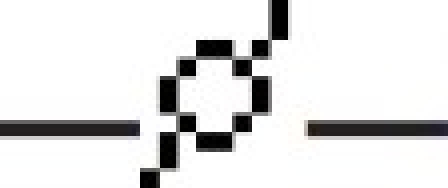
).

The batch B2 (Table 2) additionally contained intragranular fraction of Cab-O-Sil whereas batches B3-B5 additionally contained extragranular fraction of Cab-O-Sil, respectively. In batches B6-B9 (Table 2), granules of batch B1 were blended with different amount of mannitol in a mortar and pestle for 15 min. Table 3 displays the results of angle of repose and Carr's index for batches B2-B9. Captopril powder blend (batch B9) containing 12.5 mg drug was either filled in hard gelatin capsule (batch B10) of size 2 or compressed to tablet (batch B11) on a single station tablet press (Cadmach Machines Ltd., India). The capsules and tablets were characterized for *in vitro* drug release (fig. 1). Additionally, the tablets were characterized for dimension analysis, crushing strength, disintegration time, friability, content uniformity and *ex vivo* permeability study. The formulation of batch B12 was similar to batch B11 except batch B12 was prepared using 95% alcohol in place of eutectic mixture of camphor and menthol. Batch B13 tablets were prepared by direct compression after mixing of captopril, Avicel PH 102, mannitol and sodium starch glycolate with each other.

**TABLE 3:** RESULTS OF VARIOUS FORMULATED BATCHES B1-B9

Batch Code	Tests	
	
	Angle of repose^(o)^	Carr's index
B1	42.2	41.5
B2	38.8	39.9
B3	37.2	36.1
B4	35.1	32.5
B5	32.3	27.2
B6	35.4	24.8
B7	31.2	17.5
B8	26.9	13.8
B9	22.2	7.2
B12	27.2	14.1
B13	25.5	13.6

### Evaluation:

The angle of repose was measured using the fixed height funnel method[4–5]. Carr's index was measured by adding 5 g of sample from each batch to 50 ml-capacity measuring cylinder. After noting the initial volume (Vp), the cylinder was allowed to fall under its own weight onto a hard surface from a height of 2.5 cm at 2 sec intervals. The tapping was continued until no further change in volume (Vt) was noted. The Carr's compressibility index was determined using Eqn. 1[6]. Carr's index (%)= (1-Vt/Vp)×100…..(1). Tablet dimensions (diameter and thickness) and crushing strength were determined using Dr. Scheleuniger tablet hardness tester (Pharmatron 8, Solothum, Germany). Disintegration test was performed on six tablets at 37±5° in 900 ml of 0.1N hydrochloric acid (pH 1.2) using Electrolab (USP, Model 0ED-2L, Mumbai, India) disintegration tester.

Friability was evaluated as the percentage weight loss of twenty tablets tumbled in a friabilator (Electrolab, Model EF2, Mumbai, India) for 4 min at 25 rpm. The tablets were then dedusted and the loss in weight caused by fracture or abrasion was recorded as the percentage friability.

Samples of batches B10 and B11 were subjected to *in vitro* drug release for 1 h in a calibrated USP dissolution test apparatus (Electrolab, Model TDT 06-T, Mumbai, India) equipped with basket employing 900 ml 0.1N hydrochloric acid (pH 1.2). The baskets were rotated at 50 rpm and the dissolution medium was maintained at a temperature of 37±0.5° throughout the experiment[7]. Ten ml samples were withdrawn and analyzed for percentage of captopril released over time. Fresh dissolution medium was replaced after each withdrawal. Samples of *in vitro* drug release were analyzed spectrophotometrically employing Shimadzu-1700 UV/Vis spectrophotometer after suitable dilution. The absorbance of the samples was measured at a 212 nm wavelength against blank with reference to standard calibration curve obtained experimentally (r^2^=0.999)[7]. Content uniformity test was carried out as per IP 1996[7]. The amount of drug present in individual tablets was found spectrophotometrically at 212 nm after extracting the samples with 100 ml of 0.1N HCl (pH 1.2).

**Fig. 2:**
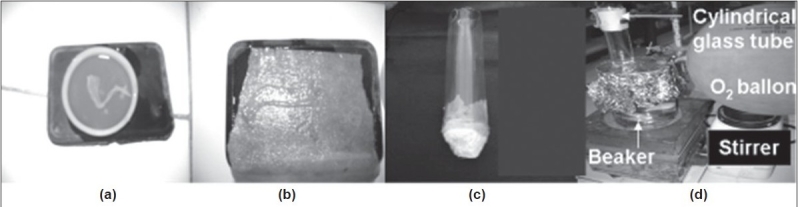
Novel modified *ex vivo* drug permeation apparatus (a) excised open sample of ileum in Petri dish, (b) excised open sample of ileum stuck on to filter paper and (c) cylindrical glass tube and (d) assembled novel modified *ex vivo* drug permeation apparatus

**Fig. 3:**
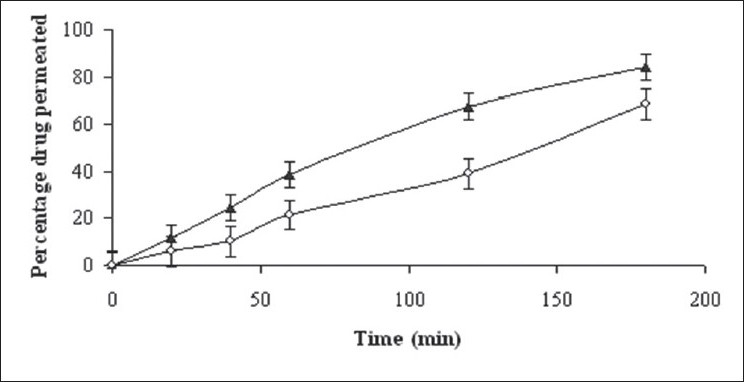
*Ex vivo* drug permeation from batches B11 and B12 Batch B11 (–▲–) and batch B12 (–◊–)

To check permeability enhancement of batches B11 and B12, *ex vivo* permeability study was carried out in a modified apparatus (fig. 2). The apparatus contained cylindrical glass tube with 31 mm internal diameter at top. The bottom of the glass tube was fused with glass cylinder with 6 mm internal diameter. Excised sample of ileum of the rat was sandwich between four nylon sieves (0.22 μm) and wrapped around cylindrical glass tube at the bottom. The cylindrical glass tube was placed in a beaker containing 400 ml Krebs ringer buffer solution maintained at 37±0.5°. The distance of glass tube from the bottom of the beaker was 17 mm. Krebs Ringer buffer solution was rotated at 50 rpm. Tablets of batches B11 or B12 were added in cylindrical glass tube containing 60 ml phosphate buffer (pH 7.2, 37±0.5°). Beaker was covered with aluminum foil and 95% pure oxygen gas was purged, using balloon, in the buffer. Ten millilitre samples were withdrawn at 20, 40, 60, 120 and 180 min from the beaker and analyzed spectrophotometrically at 212 nm against blank after suitable dilution with reference to standard calibration curve obtained experimentally (r^2^=0.995). Fresh Krebs ringer solution was replaced after each withdrawal. The results are shown in fig. 3.

**Fig. 4:**
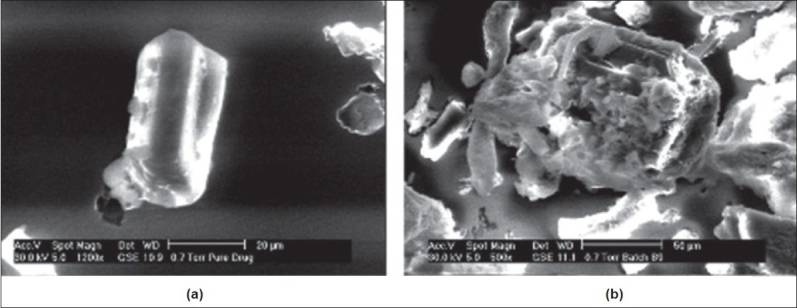
Scanning electron microscopy study (a) Pure drug and (b) batch B9

The samples of batch B9 and captopril powder were visualized with scanning electron microscope (Model ESEM TMP-EDAX, Philips, Netherlands) with EDAX (Energy dispersive analysis of X-rays) at an accelerating voltage of 30kV to evaluate captopril adsorption onto Avicel PH 102[8]. The results are displayed in fig. 4.

**Fig. 5:**
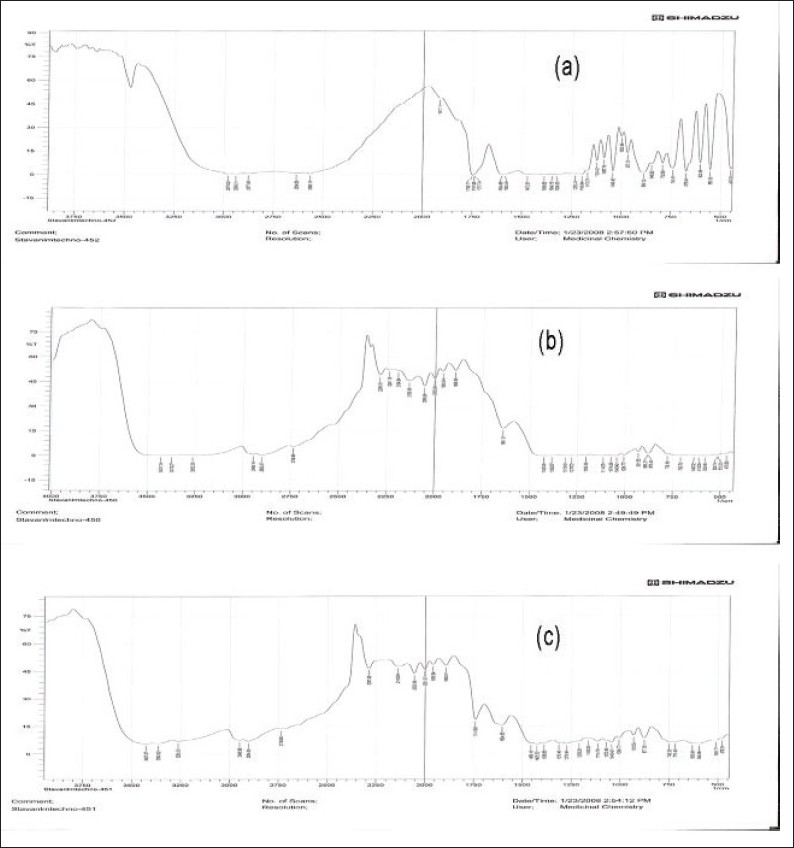
FTIR study (a) Pure drug, (b) blank formulation and (c) batch B9

Pure captopril, batch B9 with and without drug (blank) were separately mixed with IR grade potassium bromide[9]. Infrared spectra were taken using an infrared spectrophotometer (Model FTIR-8400S, Shimadzu, Japan) by scanning samples over a wave number of 4000 to 400 cm^−1^. The results are shown in fig. 5.

Short term stability study of capsules and tablets of batches B10 and B11 was carried out for 2 months at 40±2° and 75% RH by storing them in double polyethylene zip bags covered with an aluminum foil. At the end of one and two months, the samples were subjected to *in vitro* drug release. The tablets of batch B11 were additionally characterized for change in disintegration time and friability. Samples of *in vitro* drug release were analyzed by UV Spectrophotometry at 212 nm. ANOVA was applied to find any significant difference *in vitro* release drug release profile at p≤5% level of significance.

## RESULTS AND DISCUSSION

The result shown in Table 1 reveals that the undissolved solids are inversely related to the amount of menthol up to 40:60 ratio of menthol and camphor. Complete liquification of camphor and menthol was observed in case of 50:50, 60:40, 70:30, 80:20 and 90:10 of menthol and camphor at 35±2°. The eutectic blend consisting of equal proportion of camphor and menthol was used for further studies.

Solubility of captopril in eutectic mixture of camphor and menthol (batch A5) was more than 200 mg/ml. Captopril is a low dose drug (12.5 mg)[8]. Hence, adsorption of the drug solution in eutectic mixture over inert carrier was employed to overcome the problem of content uniformity. Avicel PH 102 was selected as an inert carrier considering its compressibility, flow property and self disintegration nature[10]. Polyvinylpyrrolidone (PVP K30) was employed as a binder. The results shown in Table 3 displays that the powder blend of batch B1 showed poor flow. The most widely used glidant is silicone dioxide. Cab-O-Sil, a synthetic, amorphous, untreated fumed silicon dioxide was added in subsequent batches, i.e. batches B2-B5 to resolve the issue of poor flow.

In batch B2, Cab-O-Sil was added as an intragranular fraction along with Avicel PH 102 whereas in batch B3 it was added as extragranular fraction. Angle of repose and Carr's index of batch B3 were lower (Table 3) than those of batch B2. Hence, it may be concluded that Cab-O-Sil was more efficient when added in extragranular fraction. Batch B3 containing 0.5% Cab-O-Sil failed to give excellent flow. An effort was made to improve the flow by increasing the concentration of Cab-O-Sil up to 1% (batches B4 and B5). The angle of repose and Carr's index of batches B4 and B5 were greater than 32° and 27 respectively. Hence, in subsequent batches B6-B9, co-grinding technique with mannitol was adopted. Cab-O-Sil generally is not used in pharmaceutical products at higher level.

In pharmaceutical formulations mannitol is used as a non-hygroscopic diluent (10-90% w/w)[10]. Mannitol posseses excellent compressibility and flow property. In addition, mannitol is commonly used taste masking excipient in the chewable and mouth dissolving/disintegrating tablets because of negative heat of solution, sweetness and mouth feel[10]. It is evident from the results shown in Table 3 that as the concentration of mannitol increased, the angle of repose and Carr's index decreased. Kubo and Mizobe investigated the usefulness and efficiency of the co-grinding method with D-mannitol to improve the bioavailability of a sparingly water-soluble drug, (±)-5-[[2-(2-naphthalenylmethyl)-5-benzoxazolyl]methyl]-2,4-thiazolidinedione (174), and compared it with that of the single-grinding method[11]. The co-ground mixtures showed a marked increase in the bioavailability and dissolution rate as compared with the single-ground powder. European patent describes micronization technique of slightly soluble drug by grinding drug with mannitol in a vibrational ball mill[12]. Batch B9, co-grinded with mannitol in 1:1 ratio, showed excellent flow. Hence, considering the results of angle of repose and Carr's index, batch B9 was selected for further study (preparation of capsule-batch B10 and tablet-batch B11).

The powder blend of batch B9, equivalent to one dose of captopril (167 mg), was filled in capsule (batch B10). Batch B11 was obtained by compressing 167 mg of powder blend of batch B9. The diameter and thickness of the tablet were 7.87 and 3.15 mm respectively. The crushing strength and friability of the tablet were 9±0.4 kp and 0.42%. Sodium starch glycolate and Avicel PH 102 contributed to fast disintegration (6.5 min). The moisture content in batch B9 was 1.2%. The volume of eutectic liquid formed on mixing 5 g each of camphor and menthol was 8 ml. The maximum permissible dose of camphor is 30 mg[1]. WHO acceptable daily intake of menthol, for 70 kg adult, is 28 mg[10]. Hence, the amount of camphor and menthol present in batches B10 and B11 was within permissible limits (1.4 mg each).

*In vitro* drug release study was carried out in 0.1N HCl (pH 1.2). The absorbance was measured at 212 nm[7]. The UV spectrum remained unchanged during *in vitro* study, indicating photo stability of captopril during the analytical procedure. Fig. 1 depicts that captopril was completely released from the capsules (batch B10) in 30 min. The release of captopril was faster in capsule (batch B10) than that for tablet (batch B11, 40 min). The probable reason for it could be compression force involved in preparation of tablets.

The content uniformity is critical for low dose potent drug[13]. Segregation due to particle size, or cohesiveness of an active pharmaceutical ingredient could result in significant differences in the content uniformity[14]. Rees stressed the importance of the particle size, shape, material density, moisture content, proportions of major and minor components and the sequence of incorporating constituents on homogeneity of particulate system[15]. McGinity *et al*. reported that the content uniformity and the dissolution problem of a small amount of hydrophobic drug can be resolved by preparing ordered mixes of directly compressible vehicles[16]. Nishimura and Yui studied the effect of drug affinity to the binder solution on the drug distribution in various size granules. They reported that drug that is soluble in the binder solution showed a larger variation in its distribution[17]. Miyamoto *et al*. investigated the influence of the granule size on the drug content uniformity of two model drugs and found varying effect[18]. The authors attributed these differences to the drug solubility in the binder solution. Uniform solid-solid mixing is difficult since, variables and proportion of solids, density of solids and size of solids affect the results. On the other hand, liquid can be easily mixed with solid, as done in the process of wet granulation. A layer of liquid is formed on the surface of carrier, giving uniform distribution of drug on the carrier. In the present study eutectic blend was used as a solvent and as a carrier. The idea worked well as seen in the results of content uniformity test. Tablets of batch B11 passed whereas that of batch B13 failed content uniformity test as per IP 1996 with drug content of 96±8 % and 65±10 %, respectively.

It is well known fact that solubility/dissolution is a pre-requisite to permeation of drugs through biological membrane. As the drug dissolved quickly, sufficient flux may be provided to facilitate permeation. *Ex vivo* permeation study was carried using rat ileum in a novel modified apparatus (fig. 2). From fig. 3, it is evident that batch B11, prepared using eutectic mixture of camphor and menthol, showed better permeation of drug as compared to control prepared using 95% alcohol (batch B12). Both the *ex vivo* permeability curves were dissimilar with f_2_ (similarity factor) value of 39. At the end of 3 h permeability of captopril increased by 15% as compared to control.

Scanning electron microscopy of pure drug and batch B9 confirms presence of captopril on Avicel PH 102 (fig. 4). The infrared spectra of captopril, samples of batch B9 with and without drug (blank) are shown in fig. 5. The infrared spectra of captopril and samples of batch B9 were comparable. Captopril showed two prominent peaks at 1741.6 cm^−1^ and 2565.16 cm^−1^ because of presence of carboxyl and thiol groups respectively. Blank showed absorption at 1641.31 cm^−1^, 3431.3 cm^−1^ and 1278.72 cm^−1^ because of ketone group of camphor, hydroxyl group of mannitol and ether group of Avicel PH 102, respectively. The peaks present in captopril and blank were retained in batch B9, which indicates stability of captopril during processing.

Short term stability study was carried out for 2 months at 40±2° with 75% RH. Capsules and tablets were examined for change in *in vitro* drug release. Additionally, the tablets were examined for change in disintegration time and percentage friability. The disintegration time and percentage friability of the tablets of batch B11 at the end of one and second month remained comparatively unchanged (6.2 min, 6.0 min; and 0.5, 0.63; respectively). ANOVA indicated statistical insignificant difference in the *in vitro* drug release profile from the capsules and tablets of batches B10 and B11 respectively at p≤5% with F_calculated_ < F_critical_. Hence, it was concluded that formulated capsules (batch B10) and tablets (batch B11) of captopril passed short term stability study.
